# Serum symmetric dimethylarginine concentration in healthy horses and horses with acute kidney injury

**DOI:** 10.1186/s12917-020-02621-y

**Published:** 2020-10-20

**Authors:** Natalia Siwinska, Agnieszka Zak, Malwina Slowikowska, Artur Niedzwiedz, Urszula Paslawska

**Affiliations:** 1 Department of Internal Medicine and Clinic of Diseases of Horses, Dogs and Cats, Faculty of Veterinary Medicine, Wroclaw University of Environmental and Life Sciences, C.K. Norwida 25, 50-375 Wroclaw, Poland; 2 Veterinary Institute, Faculty of Biological and Veterinary Sciences, Nicolaus Copernicus University Torun ul, Gagarina 7, 87-100 Torun, Torun, Poland

**Keywords:** SDMA range, Kidney disease, Kidney failure, Renal diagnostics, Renal biomarker

## Abstract

**Background:**

There are limited options to diagnose acute kidney injury (AKI) in horses. Symmetric dimethylarginine (SDMA) is routinely used in human and small animal medicine. The aim of this study was to assess serum SDMA concentrations in healthy horses and horses with AKI. The objective of this study was to evaluate the association of: 1) age, 2) sex, 3) body weight and 4) serum creatinine and urea levels on serum SDMA concentrations. Fifty-three healthy horses, including 17 foals (2–6 months of age) and 36 adult horses (3–29 years of age), and 23 horses with AKI were included in the study based on history, physical examination, blood analysis, urinalysis and an ultrasonographic examination of the urinary tract. Serum SDMA concentrations were measured using a non-species specific commercial ELISA test.

**Results:**

In healthy adult horses, the value of SDMA was 0.53 ± 0.14 μmol/L. The value was higher in foals (1.5 ± 0.4 μmol/L, *P* < 0.001). Horses with AKI had significantly higher concentrations of SDMA compared to healthy horses (1.76 ± 1.05 μmol/L, *P* < 0.001). In the healthy adult horses, there was no association of sex, age or body weight on SDMA. However, a significant positive relationship was found between serum creatinine and SDMA concentrations.

**Conclusions:**

Healthy adult horses had SDMA values similar to those of other species. Foals had higher SDMA values. Therefore, different reference values should be created for them. The study confirmed an increased SDMA in horses with AKI. This, as well as the low influence of extrarenal factors on the SDMA values, may confirm its usefulness in the diagnosis of kidney dysfunction. Higher SDMA values may also indicate a more advanced degree of kidney dysfunction. Further research is required to determine whether SDMA could be used to detect kidney dysfunction in the asymptomatic stage of AKI.

**Supplementary information:**

The online version contains supplementary material available at 10.1186/s12917-020-02621-y.

## Background

In contrast to studies on dogs and cats, there is scarce literature on the prevalence of kidney diseases, including acute kidney injury (AKI) in the general equine population. However, there are several reports of AKI in horses, with increasing prevalence in intensive care units [1–3]. Currently, kidney disease in horses is diagnosed based on increased serum creatinine and urea levels, abnormal urinalysis and ultrasonographic lesions detected in kidneys. The measurement of serum creatinine or the endogenous creatinine clearance may be used to assess the GRF in animals. However, creatinine levels can be influenced by numerous extrarenal factors [4, 5]. Previous studies have also used other parameters, such as the ratio of urine and plasma urea nitrogen, fractional excretion of sodium (FENa) and γ-glutamyl transpeptidase (GGT) to creatinine ratio in urine, to assess renal function or injury [6, 7]. Due to the increase in biomarker demand, there is a growing interest in new AKI biomarkers in horses. However, the number of biomarker studies conducted in horses is limited [8–10].

Symmetric dimethylarginine (SDMA), a new biomarker for the early detection of kidney dysfunction, is routinely used in human and small animal medicine. SDMA is an endogenous methylated form of the arginine that is released into circulation during normal protein catabolism [11]. Ninety percent of SDMA is excreted by the kidneys into urine in an unchanged form [12–14]. Hence, this metabolite accumulates in the course of renal dysfunction [15]. Also, there are only a few non-renal factors that affect the SDMA concentration, which to date have not been studied in horses. A stronger correlation was found between SDMA and GFR than between serum creatinine and GFR [16]. Besides, there was a significantly earlier increase in the concentration of serum SDMA than in serum creatinine in animals with progressing kidney dysfunction [4, 17, 18]. To the authors’ knowledge, only one study has been published assessing normal SDMA values in healthy, adult draft horses and two studies in neonatal foals (with normal creatinine value and with hypercreatininemia) [19–21]. Therefore, this is the first study comparing SDMA values in healthy horses and horses with AKI.

The objectives of the study were to 1) determine normal values of SDMA for adult horses and foals, 2) determine values of SDMA in horses with AKI and compare them to those in healthy horses, 3) assess the association of age, sex and body weight with SDMA, 4) compare and correlate SDMA to serum creatinine and urea.

## Results

The results of the serum creatinine, urea, and SDMA concentrations in the studied horses are presented in Table 1.

**Table 1:** Serum creatinine, urea, and symmetric dimethylarginine (SDMA) concentrations in the studied horses

Group		*n*	Results	Creatinine (μmol/L)	Urea (mmol/L)	SDMA (μmol/L)
Group I – healthy horses	Foals	17	min.	79.0	2.8	0.75
			q1 – median – q3	101.0–111.0 – 121.0	3.3–3.7 – 4.6	1.3–1.5 – 1.75
			max.	159.0	5.2	2.18
			lowerCl	103.6	3.5	1.29
			upperCl	121.1	4.3	1.71
	Adults	36	min.	69.0	3.2	0.1
			q1 – median – q3	101.25–114.5 – 133.0	4.0–5.2* - 5.8	0.47–0.54* - 0.65
			max.	156.0	6.6	0.71
			lowerCl	107.1	4.7	0.48
			upperCl	122.8	5.4	0.58
Group II - horses with AKI		23	min.	161.7	7.7	0.75
			q1 – median – q3	186.8–208.7^# - 407.5	10.2–13.1^# - 20.6	0.95–1.6^ - 2.19
			max.	1032.5	104.7	4.55
			lowerCl	229.2	11.1	1.31
			upperCl	405.9	29.7	2.21

*Note: AKI* acute kidney injury, * significant differences *P* < 0.05 between foals and healthy adults from group I; # significant differences *P* < 0.05 between foals and group II; ^ significant differences *P* < 0.05 between healthy adults and group II

The mean SDMA values for group I were: 0.53 SD 0.14 μmol/L for adults and 1.5 SD 0.41 μmol/L for foals. In foals, the SDMA values were higher than those in healthy adult horses (*P* < 0.001). The mean serum concentration of SDMA in the group II was 1.76 SD 1.05 μmol/L and it was significantly increased in comparison to the adult horses from group I (P < 0.001). There were no statistically significant differences in those values between foals form group I and horses from group II.

Correlations between SDMA and other parameters are presented in Table 2. There was no differences between the concentration of SDMA in males and females in any of the groups.

**Table 2:** Spearman correlation between the SDMA and age, body weight, serum creatinine and urea in the examined horse groups

	SDMA correlations		
	Group I – healthy horses		Group II - horses with AKI
	Foals	Adults	
Age	r_s_ = −0.51	NS	NS
Body weight	r_s_ = −0.55	NS	NS
Serum creatinine	NS	r_s_ = 0.36	r_s_ = 0.71
Serum urea	NS	NS	r_s_ = 0.81

*Note: AKI* acute kidney injury, *r*_*s*_ Spearman correlation coefficient; *NS* nonsignificant. All correlations presented were significant with *P* < 0.05

The multivariate analysis also revealed significantly higher concentrations of SDMA in foals from group I and group II than in adults from group I. It further revealed significant positive correlations between serum creatinine and SDMA, as well as between urea and SDMA. An additional model with the interaction term for age and individual groups revealed that older animals with AKI had lower SDMA concentrations. No such correlation was seen in the remaining groups.

There was no significant difference in the sex distribution between the studied groups. The mean age of the adults from group I and group II did not differ significantly. Group II had a significantly lower body weight than the adults from group I (*P* = 0.04). There were no significant differences in the serum creatinine values between foals and adults from group I. In group I, foals had significantly lower urea values compared to adult horses (*P* < 0.001). There was no correlation between body weight and the concentration of urea and serum creatinine between the groups.

## Discussion

This is the first study presenting values of SDMA for healthy adult horses and foals as well as values of SDMA in horses with AKI. Additionally, the authors assessed the impact of age, sex, and body weight on the concentration of this parameter in this species and correlated the SDMA values with serum creatinine and urea values.

The obtained SDMA values in blood serum in healthy adult horses correspond to the results obtained by Schott et al. (2018) in draft horses and are similar to the reference values in humans (from 0.3 to 0.7 μmol/L), dogs (from 0.28 to 0.58 μmol/L) and cats (from 0.35 to 0.6 μmol/L) [4, 19, 22–25].

In the present study, healthy foals had a higher mean SDMA concentration than the healthy adult horses. These results are consistent with those obtained in the studies by Bozorgmanesh et al. carried out at neonatal foals [20, 21]. A similar finding was noted in humans, where children had significantly higher SDMA concentrations compared to adults and the elderly [26]. It seems unlikely that an increased SDMA concentration in young animals is associated with inadequate excretion. The cause and mechanism of this increase are currently unknown, but it is postulated that the increased number of processes, such as protein arginine methylation in signal transduction, mRNA splicing, transcriptional control, DNA repair and protein translocation in growing animals may be associated with this increase [27]. In dogs SDMA significantly decreased upon animals reaching maturity [17]. Similarly, in this study, older foals had lower SDMA values than younger foals and this result was in agreement with the observations made in the studies with neonatal foals [20, 21]. Determining the exact age cut-off point of the SDMA value in foals equal to that of adult horses requires further testing. The current study assessed too few animals between six months and three years of age to allow conclusions to be drawn.

The range of SDMA concentrations in horses with AKI was similar to results in humans (the SDMA concentration in people with acute kidney failure and chronic kidney disease (CKD) 1.02–4.6 μmol/L), dogs (AKI 0.39 - > 5.83 μmol/L, CKD 0.58 - > 4.83 μmol/L) and cats (CKD 0.67 - > 5.55 μmol/L), where an increase in SDMA concentration corresponded to disease severity [4, 12, 25, 28]. Similarly, in our study, all the horses with AKI had significantly higher SDMA values than the adult horses from group I. Also, none of the animals with azotaemia had low SDMA values, and none of the healthy adult horses had high SDMA levels. In the presented population, SDMA correctly distinguished the ill individuals. This indicates that SDMA may be of diagnostic value for the confirmation of kidney dysfunction in horses. More studies are needed, especially for horses at risk of AKI without elevation of serum creatinine, to determine if SDMA is superior to creatinine in detecting renal dysfunction.

In healthy adult humans, there was a positive correlation between SDMA and age [29]. A similar finding was observed in cats, where serum SDMA increased in cats older than 15 years of age [30]. In the healthy adult horses, an age-related increase in SDMA concentration was not observed, possibly due to a low number of aged horses in the group. In a study of dogs with CKD, SDMA concentrations increased when the GFR decreased by as little as 30% [17]. In the horses studied, such a correlation could not be determined, as GFR was not measured.

Studies in healthy humans and small animals showed minimal or no effect of sex or body weight and mass on the concentrations of SDMA [24, 30–33]. A lack of influence of extrarenal factors on SDMA is another advantage of this compound over serum creatinine as a kidney dysfunction biomarker. In all examined horses, SDMA was gender independent. A correlation between SDMA and body weight was observed in foals. This is most likely not associated with body weight, but rather with a decrease of the high post-natal values to lower adult values.

A positive correlation between the concentration of SDMA and serum creatinine, as well as SDMA and serum urea was confirmed in the group of horses with AKI. The results indicate that the concentration of SDMA increases as kidney dysfunction progresses. Considering the potential use of SDMA for detecting subclinical renal dysfunction, the group of healthy adult horses was studied meticulously. In that group, a positive correlation between the concentration of SDMA and serum creatinine was found, while there was no correlation between SDMA and urea. Similar results were obtained in healthy humans and people with subclinical kidney disease [26]. This may be explained by the variability of the urea concentration, which is affected by several extrarenal factors. The finding of no correlation between the values of SDMA and serum creatinine in the group of foals correspond to findings in juvenile dogs [17].

The diagnostic accuracy of a biomarker may differ depending on the duration of kidney disease or its aetiology. It should also be mentioned that some biomarkers assess organ function, while others evaluate tissue damage. Each biomarker also has its own specific concentration-time curve [34]. These factors were not assessed in the present study as GFR was not measured and SDMA concentrations were evaluated only once in each horse. It would be interesting to monitor SDMA changes in individual horses during the progression or resolution of AKI. The absence of a baseline serum creatinine concentration may have underestimated the severity kidney dysfunction in the examined horses. Additionally, the concentration of SDMA was not measured before the appearance of clinical signs. Both of the above could not entirely exclude some pre-existing loss of renal function that could have been exacerbated by a new acute disease process. The current study did not investigate the impact of diet and breed on the SDMA concentration due to the large variety of animals. However, these factors could have had some effect on the results. The final limitation was an absence of a group of neonatal foals and foals with kidney dysfunction. Further study of larger cohorts of horses are needed to establish the exact reference values of SDMA in this species. Before a new biomarker of renal impairment is introduced, its advantage over current diagnostic methods (such as serum creatinine) in identifying AKI needs to be demonstrated. Due to the selection of the group, the studies performed do not provide sufficient evidence that SDMA may be a better biomarker of kidney dysfunction than currently used methods. Therefore, more research is warranted.

## Conclusion

The study presents SDMA values for healthy horses of different ages as well as for horses with a different stage of AKI. SDMA values in healthy adult horses were similar to those in other animal species. SDMA concentrations in healthy foals were significantly higher than in healthy adult horses. SDMA seems to be a promising biomarker of kidney dysfunction in horses because of its distinctly different values between healthy adult horses and those with azotaemia. SDMA may be useful in clinical scenarios where a suspicion of kidney dysfunction needs to be confirmed. Horses with a higher serum creatinine value had higher SDMA values, which indicates that SDMA concentration may indicate the stage of renal dysfunction. Furthermore, SDMA seems to be less susceptible than creatinine to the influence of extrarenal parameters. Further studies are required to determine whether SDMA may be used to detect asymptomatic renal dysfunction in horses.

## Methods

Serum SDMA values were measured in 76 horses (mean age 10.3 SD 9.1 years, mean body weight 432.3 SD 143.7 kg) of various breeds and genders. The animals were divided into groups. Group I consisted of healthy animals that underwent routine clinical examinations and were included in the study. That group was split into two subgroups. The first subgroup included 17 foals (8 females and 9 males) from 2 to 6 months old (mean 3.9 SD 1.2 months) weighing 195.8 SD 27.9 kg, and the second subgroup included 36 adult horses (18 females and 18 males) from 3 to 29 years old (mean 14.7 SD 9 years) with a mean weight of 515.2 SD 73.7 kg. The inclusion criteria were as follows: a good body condition (rated as 4–5 on a 9-point scale [35]), no history of and no existing systemic or local disease for at least a year before the study, no suspected or diagnosed urinary tract diseases, normal haematological and biochemical blood analysis and urinalysis, no urinary tract lesions visible on ultrasonography as well as no disease six months after collecting the study material. None of the horses included in the healthy group was treated with potentially nephrotoxic drugs. Additional inclusion criteria were adopted for foals and included: no history of disease in the mares, normal pregnancy and delivery of the foal, normal placental expulsion and normal foal growth without disorders. Group II consisted of 23 adult horses (12 females and 11 males; aged 10.7 SD 6.7 years; weight 477.4 SD 67.7 kg) that developed acute kidney dysfunction (acute kidney injury and failure) secondary to other medical problems, such as gastrointestinal disorders (e.g., peritonitis, small or large bowel diseases – inflammation, impaction, displacement or torsion), prolonged surgery, use of potentially nephrotoxic drugs, a systemic inflammatory response (SIRS) and other unspecified reasons. The inclusion criteria for group II were acute disease accompanied by azotaemia (increase in serum creatinine and urea levels above the reference range). The exclusion criteria included a history of urinary tract dysfunction or disease, administering potentially nephrotoxic drugs within a few months before the study (with the exception of drugs used to treat the current primary disease), elevated serum creatinine and urea without other signs of the primary disease.

In all horses, a physical examination was performed, and the following parameters were measured: body weight using electronic scales, blood analysis (hematology and biochemistry: serum creatinine, urea, aspartate aminotransferase, alanine aminotransferase, γ-glutamyl transpeptidase, alkaline phosphatase, creatine kinase, total protein, albumin, glucose, sodium, potassium, chloride, magnesium, calcium), a complete urinalysis with a sediment examination and a transabdominal and rectal (except foals) ultrasonographic examination of the urinary tract. In all animals (and at admission for group II horses [within 48 h of the onset of clinical signs of disease]), venous blood was collected from the external jugular vein into clot activator tubes, which were then centrifuged at 4000 RPM for 10 min. (1433 relative centrifuge force) in the MPW-250 laboratory centrifuge (MPW Med. Instruments, Warsaw, Poland). The obtained serum was cooled and transported to a laboratory to measure the levels of serum creatinine, urea, and SDMA. The serum creatinine and urea concentrations were determined using the AU680 chemistry analyser (Beckman Coulter, California, USA) with dedicated reagents. The physiological values for horses were determined by a reference laboratory and ranged from 71.0 to 159.0 μmol/L for serum creatinine and from 3.30 to 6.70 mmol/L for urea. To measure SDMA, the Labor der SYNLAB vet GmHb (Ausburg, Germany) laboratory used a commercially available and non-species specific enzyme immunoassay ELISA kit (DLD Diagnostika GmbH, Hamburg, Germany). The test validation information is provided in the Supplementary Materials (Suppl. 1). SDMA measurements using ELISA were repeated thrice. The average of the triplicate values was used in statistical analysis.

All the examinations performed in this study were routine and non-invasive. Following the existing law applicable in the Experiments on Animals Act from January 15th 2015 (Journal of Laws of the Republic of Poland, 2015, item. 266), non-invasive clinical studies do not require ethical approval. The owners of the horses consented to the research and the publication of its results.

### Statistical analysis

The statistical analysis was carried out using R for Windows (version 3.5) [36]. The data were presented as mean, standard deviation, median, and quartiles. Due to non-normal data distribution, nonparametric statistical tests were used where appropriate. The analysis of the sex distribution between the groups was carried out using Fisher’s exact test. The analysis of the differences in the quantitative variables (body weight, age, urea, serum creatinine, and SDMA concentrations) between the groups was performed using the Kruskal-Wallis test with a post-hoc analysis done using the Conover method with the Holm adjustment. Correlations of variables, such as body weight and the concentration of urea and serum creatinine, as well as age, body weight, urea, serum creatinine, and SDMA concentrations were assessed using the Spearman correlation coefficient. Differences in SDMA concentrations between males and females were evaluated by the Mann-Whitney test. Multivariate regression modelling was performed to evaluate independent effect predictors such as age, sex, body weight, serum urea and creatinine on concentrations of SDMA. In addition to the model assessing the main effects, a second model was constructed to evaluate the interaction terms between age and the groups. Since the group II contained only adult horses, a detailed analysis of sick and healthy animals was carried out only on adults. The significance level was set at 5%.

## Supplementary information

Additional file 1. Supplementary Material 1. Validation of ELISA test. (DOCX 41 kb)

## Data Availability

The datasets during and/or analysed during the current study available from the corresponding author on reasonable request.

## Abbreviations

AKI: Acute kidney injury
GFR: Glomerular filtration rate
CKD: Chronic kidney disease
SDMA: Symmetric dimethylarginine

## References

[1] Savage VL, Marr CM, Bailey M, Smith S (2019). Prevalence of acute kidney injury in a population of hospitalized horses. J Vet Intern Med.

[2] Groover ES, Woolums AW, Cole DJ, LeRoy BE (2006). Risk factors associated with renal insufficiency in horses with primary gastrointestinal disease: 26 cases (2000-2003). JAVMA.

[3] Chaney KP, Holcombe SJ, Schott HC, Barr BS (2010). Spurious hypercreatininemia: 28 neonatal foals (2000-2008). J Vet Emerg Crit Care.

[4] Hall JA, Yerramilli M, Obare E, Yerramilli M, Jewell DE (2014). Comparison of serum concentrations of symmetric dimethylarginine and creatinine as kidney function biomarkers in cats with chronic kidney disease. J Vet Intern Med.

[5] Polzin DJ (2013). Evidence-based step-wise approach to managing chronic kidney disease in dogs and cats. J Vet Emerg Crit.

[6] Grossman BS, Brobst DF, Kramer JW, Bayly WM, Reed SM (1982). Urinary indices for differentiation of prerenal azotemia and renal azotemia in horses. JAVMA.

[7] El-Ashker MR, Hussein HS, El-Sebaei MG (2012). Evaluation of urinary variables as diagnostic indicators of acute kidney injury in Egyptian draft horses treated with phenylbutazone therapy. J Eq Vet Sci.

[8] Arosalo BM, Raekallio M, Rajamäki M, Holopainen E, Kastevaara T, Salonen H, Sankari S (2007). Detecting early kidney damage in horses with colic by measuring matrix metalloproteinase −9 and −2, other enzymes, urinary glucose and total proteins. Acta Vet Scand.

[9] El-Ashker MR (2011). Acute kidney injury mediated by oxidative stress in Egyptian horses with exertional rhabdomyolysis. Vet Res Commun.

[10] Jacobsen S, Berg LC, Tvermose E, Laurberg MB, van Galen G (2018). Validation of an ELISA for detection of neutrophil gelatinase-associated lipocalin (NGAL) in equine serum. Vet Clin Pathol.

[11] Paltrinieri S, Giraldi M, Prolo A (2018). Serum symmetric dimethylarginine and creatinine in Birman cats compared with cats of other breeds. J Feline Med Surg.

[12] Schwedhelm E, Boger RH (2011). The role of asymmetric and symmetric dimethylarginines in renal disease. Nat Rev Nephrol.

[13] Nijveldt RJ, Teerlink T, Van Guldener D (2003). Handling of asymmetrical dimethylarginine and symmetrical dimethylarginine by the rat kidney under basal conditions and during endotoxemia. Nephrol Dial Transplant.

[14] Kielstein JT, Boger RH, Bode-Boger SM (2002). Marked increase of asymmetric dimethylarginine in patients with incipient primary chronic renal disease. J Am Soc Nephrol.

[15] Vallance P, Leone A, Calver A, Collier J, Moncada S (1992). Accumulation of an endogenous inhibitor of nitric oxide synthesis in chronic renal failure. Lancet..

[16] Braff J, Obare E, Yerramilli M, Elliott J, Yerramilli M (2014). Relationship between serum symmetric dimethylarginine concentration and glomerular filtration rate in cats. J Vet Intern Med.

[17] Nabity MB, Lees GE, Boggess MM (2014). Symmetric dimethylarginine assay validation, stability, and evaluation as a marker for the early detection of chronic kidney disease in dogs. J Vet Intern Med.

[18] Hall JA, Yerramilli M, Obare E, et al. Serum concentrations of symmetric dimethylarginine and creatinine in cats with kidney stones. PLoS One. 2017. 10.1371/journal.pone.0174854. 10.1371/journal.pone.0174854 PMC5383095 28384169

[19] Schott HC, Gallant L, Coyne M. Symmetric dimethylarginine and creatinine concentrations in draft horse breeds. J Vet Intern Med. 2018;32:2128–2129 [abstract]. 10.1111/jvim.16042 PMC7995414 33543506

[20] Bozorgmanesh R, Coyne M, Murphy R, Halliwell E, Coyne M, Murphey R, Hegarty E, Slovis N. Equine neonatal symmetric dimethylarginine (SDMA): results of two pilot studies. J Vet Intern Med. 2019; 33:2440–2441 [abstract].

[21] Bozorgmanesh R, Magdesian G, Offer K, Hegarty E, Slovis N. Equine neonatal symmetric dimethylarginine in sick neonates with hypercreatininemia. J Vet Emerg Crit Care. 2019;29:S30 [abstract].

[22] Torino C, Pizzini P, Cutrupi S, et al. Vitamin D and methylarginines in chronic kidney disease (CKD). PLoS One. 2017. 10.1371/journal.pone.0185449. 10.1371/journal.pone.0185449 PMC5627906 28976989

[23] Martens-Lobenhoffer J, Bode-Böger SM (2006). Fast and efficient determination of arginine, symmetric dimethylarginine, and asymmetric dimethylarginine in biological fluids by hydrophilic-interaction liquid chromatography-electrospray tandem mass spectrometry. Clin Chem.

[24] Fleck C, Schweitzer F, Karge E, Busch M, Stein G (2003). Serum concentrations of asymmetric (ADMA) and symmetric (SDMA) dimethylarginine in patients with chronic kidney diseases. Clin Chim Acta.

[25] Dahlem DP, Neiger R, Schweighauser A (2017). Plasma symmetric dimethylarginine concentration in dogs with acute kidney injury and chronic kidney disease. J Vet Intern Med.

[26] Protas TP, Tenderenda-Banasiuk E, Taranta-Janusz K (2014). Is symmetric dimethyarginine a sensitive biomarker of subclinical kidney injury in children born with low birth weight?. Biomarkers..

[27] Bedford MT, Clarke SG (2009). Protein arginine methylation in mammals: who, what, and why. Mol Cell.

[28] Jepson RE, Syme HM, Vallance C, Elliott J (2008). Plasma asymmetric dimethylarginine, symmetric dimethylarginine, L-arginine, and nitrite/nitrate concentrations in cats with chronic kidney disease and hypertension. J Vet Intern Med.

[29] Schwedhelm E, Xanthakis V, Maas R (2011). Plasma symmetric dimethylarginine reference limits from Framingham offspring cohort. Clin Chem Lab Med.

[30] Hall JA, Yerramilli M, Obare E (2014). Comparison of serum concentrations of symmetric dimethylarginine and creatinine as kidney function biomarkers in healthy geriatric cats fed reduced protein foods enriched with fish oil, L-carnitine, and medium-chain triglycerides. Vet J.

[31] Hall JA, Yerramilli M, Obare E (2015). Relationship between lean body mass and serum renal biomarkers in healthy dogs. J Vet Intern Med.

[32] Moesgaard SG, Holte AV, Mogense T (2007). Effect of breed, gender, exercise and white-coat effect on markers of endothelial function in dogs. Res Vet Sci.

[33] Hokamp JA, Nabity MB (2016). Renal biomarkers in domestic species. Vet Clin Pathol.

[34] Kielstein JT, Veldink H, Martens-Lobenhoffer J (2011). SDMA is an early marker of change in GFR after living-related kidney donation. Nephrol Dial Transplant.

[35] Henneke DR, Potter GD, Kreider JL, Yeates BF (1983). Relationship between condition score, physical measurements and body fat percentage in mares. Equine Vet J.

[36] R Core Team. R: A language and environment for statistical computing. R Foundation for Statistical Computing, Vienna, Austria. 2018; URL https://www.R-project.org/.

