# Supplementary material for: Serum symmetric dimethylarginine concentration in healthy horses and horses with acute kidney injury
Source: BMC Vet Res. 2020 Oct 20;16:396. doi: 10.1186/s12917-020-02621-y (PMC7576750; doi:10.1186/s12917-020-02621-y)

**Supplementary Material 1.**

**Validation of ELISA test**

The experiment scheme included the validation of the SDMA assay. In order to check the precision and repeatability of the assay test results, the coefficients of variability (CV) were established. The intraassay (within run precision), the interassay (run to run precision) and linearity experiments were performed. The results for ten repeated runs for each sample showed coefficients of variation of 13.46% (sample 1) and 13.57% (sample 2) for intraassay (Table 1) and 14.0% (sample 1) and 10.48% (sample 2) for the interassay (Table 2) tests. In the linearity experiment, the samples from a patient pool were assayed under various known dilutions (see scheme below). The results are reported in a table (Table 3) and a diagram (Fig. 1). The bias was always under 15%. The coefficient of determination was 0.9676.

**Table 1. Results of the intraassay accuracy test run in two pools of patient serum samples of higher mean (sample ES1) and lower mean (sample ES2) SDMA concentration.**

| **Sample ES1:**  Repetition no. | SDMA value (μmol/l) | **Sample ES2:**  Repetition no. | SDMA value (μmol/l) | |
| --- | --- | --- | --- | --- |
| 1 | 2.23 | 1 | 0.44 | |
| 2 | 1.60 | 2 | 0.43 | |
| 3 | 1.79 | 3 | 0.50 | |
| 4 | 2.19 | 4 | 0.53 | |
| 5 | 2.09 | 5 | 0.66 | |
| 6 | 1.95 | 6 | 0.60 | |
| 7 | 1.72 | 7 | 0.50 | |
| 8 | 1.67 | 8 | 0.57 | |
| 9 | 1.59 | 9 | 0.60 | |
| 10 | - | 10 | 0.55 | |
|  | | | | |
| Test samples | **n** | **x** | **± S** | **CV** |
| **Sample ES1:**  Equine serum  (patient pool) – higher SDMA values | 9 | 1.87 | 0.252 | 13.46 |
| **Sample ES2:**  Equine serum  (patient pool) – lower SDMA values | 10 | 0.54 | 0.073 | 13.57 |

***Samples 1 and 2 (pooled equine serums of different mean SDMA values) run in repetitions; n - number of repetitions; x - mean SDMA value; ± S - standard deviation; CV - coefficient of variation**

**Table 2. Results of the interassay accuracy test run in two control materials of a lower mean (sample CM1) and higher mean (sample CM2) SDMA concentration.**

| **Sample CM1:**  Repetition no. | SDMA value (μmol/l) | **Sample CM2:**  Repetition no. | SDMA value (μmol/l) | |
| --- | --- | --- | --- | --- |
| 1 | 0.42 | 1 | 0.72 | |
| 2 | 0.39 | 2 | 0.99 | |
| 3 | 0.46 | 3 | 0.90 | |
| 4 | 0.60 | 4 | 0.91 | |
| 5 | 0.54 | 5 | 0.80 | |
| 6 | 0.54 | 6 | 0.83 | |
| 7 | 0.42 | 7 | 0.70 | |
| 8 | 0.48 | 8 | 0.80 | |
| 9 | 0.47 | 9 | 0.82 | |
| 10 | 0.55 | 10 | 0.86 | |
|  | | | | |
| Test samples | **n** | **x** | **± S** | **CV** |
| **Sample CM1:**  Control material – lower SDMA values | 10 | 0.49 | 0.068 | 14.00 |
| **Sample CM2:**  Control material – higher SDMA values | 10 | 0.83 | 0.087 | 10.48 |

***Samples 1 and 2 (control materials of different SDMA values) run in repetitions; n - number of repetitions; x - mean SDMA value; ± S - standard deviation; CV - coefficient of variation**

**Dilution scheme for linearity experiment (H – equine serum of high SDMA value; L – equine serum of low SDMA value):**

Level 1 = 10 parts H + 0 parts L (H undiluted)

Level 2 = 9 parts H + 1 parts L

Level 3 = 8 parts H + 2 parts L

Level 4 = 7 parts H + 3 parts L

Level 5 = 6 parts H + 4 parts L

Level 6 = 5 parts H + 5 parts L

Level 7 = 4 parts H + 6 parts L

Level 8 = 3 parts H + 7 parts L

Level 9 = 2 parts H + 8 parts L

Level 10 = 1 parts H + 9 parts L

Level 11 = 0 parts H + 10 parts L (L undiluted)

| **Table 3. Linearity experiment results.** | | | |  |  |  |  |  |  |
| --- | --- | --- | --- | --- | --- | --- | --- | --- | --- |
|  | |  |  |  |  |  |  |  |  |
| Parameter | | SDMA |  |  | Manufacturer: | DLD, Hamburg, Germany |  |  |  |
|  | |  |  |  | Method: | ELISA |  |  |  |
|  | |  |  |  |  |  |  |  |  |
| Test Range according to manufacturer | | 0.2 µmol/l | 3 µmol/l |  | |  |  |  |  |
|  | |  |  |  | Laboratory: | synlab.vet GmbH |  | |  |
| Sample | | SDMA concentration | unit |  |  | Standort Augsburg |  | |  |
| Low N | | 0.49 | µmol/l |  |  | Gubener Str. 39 |  | |  |
| High H | | 1.53 | µmol/l |  |  | 86156 Augsburg, Germany |  | |  |
|  | |  |  |  |  |  |  |  |  |
|  | |  |  |  |  |  |  |  |  |
| Dilution level # | | Volume L (µl) | Volume H (µl) | Expected SDMA value (µmol/l) | Measured SDMA value  (µmol/l) | Bias% |  |  |  |
| 1 | | 0 | 400 | 1.53 | 1.53 | 0.00 |  |  |  |
| 2 | | 40 | 360 | 1.426 | 1.49 | 4.49 |  |  |  |
| 3 | | 80 | 320 | 1.322 | 1.52 | 14.98 |  |  |  |
| 4 | | 120 | 280 | 1.218 | 1.25 | 2.63 |  |  |  |
| 5 | | 160 | 240 | 1.114 | 1.14 | 2.33 |  |  |  |
| 6 | | 200 | 200 | 1.01 | 1.12 | 10.89 |  |  |  |
| 7 | | 240 | 160 | 0.906 | 0.88 | -2.87 |  |  |  |
| 8 | | 280 | 120 | 0.802 | 0.7 | -12.72 |  |  |  |
| 9 | | 320 | 80 | 0.698 | 0.68 | -2.58 |  |  |  |
| 10 | | 360 | 40 | 0.594 | 0.61 | 2.69 |  |  |  |
| 11 | | 400 | 0 | 0.49 | 0.49 | 0.00 |  |  |  |
|  |  |  |  |  |  |  |  |  |  |

***Samples L (low SDMA value) and H (high SDMA value) assayed in dilutions as described above.**

**Fig. 1. Diagram of the linearity experiment results.**

**Dots represent expected SDMA values (X-axis) compared to measured SDMA values (Y-axis) in subsequent dilutions (see Table 3).**

**R^2^ – coefficient of determination**


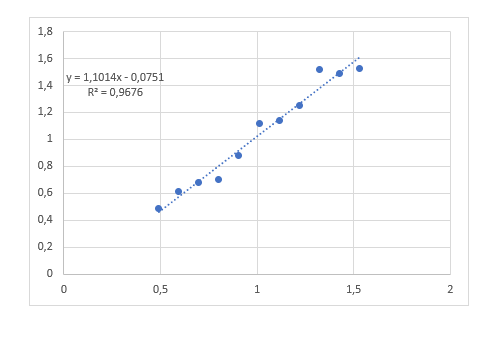

Supplement: Supplementary file 1 — Supplementary Material 1. Validation of ELISA test. (DOCX 41 kb) [file 12917_2020_2621_MOESM1_ESM.docx]
